# Nitrous oxide during anesthesia for laparoscopic donor nephrectomy: Does it matter?

**Published:** 2008

**Authors:** Pratipal Singh, Manu Gupta, Aneesh Srivastava

**Affiliations:** Department of Urology and Renal Transplantation, Sanjay Gandhi Post Graduate Institute of Medical Sciences, Lucknow - 226 014, India. E-mail: aneesh@sgpgi.ac.in

## SUMMARY

Nitrous oxide (N_2_ O) remains a popular anesthetic agent because it is inexpensive, analgesic and short-acting. It is most widely used mixed with oxygen to reduce the concentration of other potent volatile anesthetics to minimize cardiorespiratory depression. Use of N_2_ O to anesthetize patients during laparoscopic surgery has been a matter of debate between anesthesiologists and surgeons worldwide. It has been suggested that it causes bowel distension because N_2_ O can diffuse in the bowel lumen. When bowel distension occurs in the course of an operation it may increase surgical difficulty to a considerable degree.

The authors randomized laparoscopic kidney donors into two groups. Group I received N_2_ O and oxygen inhalation through anesthesia and Group II received a mixture of air and oxygen. All patients received the same pre-anesthetic and anesthetic medications. The surgeon was blinded to the use of N_2_ O. The surgeon was given the option to discontinue N_2_ O use (if it was used) if he/she thought that the bowel distension was increasing surgical risk. Postoperative data were collected on bowel symptoms, pain and recovery. A total of 28 patients were enrolled in the study, 12 received N_2_ O (Group I) and 16 did not receive N_2_ O (Group II). Mild to moderate bowel distention was reported by the surgeons in six patients in Group I and one patient only in Group II (50% *vs.* 6%, *P* = 0.007). Severe bowel distention was encountered in four patients, three of whom received N_2_ O (25% of Group I). Nausea and vomiting on postoperative Day 1 was reported by 50% of patients in Group I and 25% of Group II. There was no difference in the pain scores between the two groups. The authors concluded that the use of N_2_ O anesthetic causes bowel distension in 50% of abdominal laparoscopic donor nephrectomy operations. The distension was severe enough to interfere with the progress of surgery in 25% of cases and the use of N_2_ O had to be discontinued.

## COMMENTS

Despite its long track record of providing good analgesic effect and thus helping to reduce the concentration of other anesthetics, controversies have surrounded the use of N_2_ O. First its role in postoperative nausea and vomiting (PONV), secondly its adverse effects related to absorption and expansion of air-filled spaces like bowel and middle ear, thirdly its potential toxic effects on cell function via inactivation of vitamin B12 and lastly its effect on embryonic development.[1] The first two concerns are most valuable in clinical practice. N_2_ O is relatively insoluble in blood and diffuses out into the bowel till the partial pressure equals, hence higher the inspired concentration of N_2_ O, higher the partial pressure required to reach the equilibrium and more the distension of bowel. However, with 70% N_2_ O only a threefold increase in intestinal gas volume is theoretically possible. Normal bowel contains less than 100 ml of air so the maximum increase in volume would be 233 ml, hardly enough to interfere significantly with most procedures.[2] Probably due to these contradictory evidences a few studies failed to show any effect of N_2_ O on bowel distension in patient undergoing laparoscopic surgery.[3] Clinical effect of N_2_ O diffusion into intraperitoneal carbon dioxide pneumoperitoneum remains unclear. However, a recent report advised against its use to minimize the effects of gas embolism if it happens at all during laparoscopic donor nephrectomy.[4]

Because severe distention occurred in one patient in the non N_2_ O group, factors other than N_2_ O must also be at work in the present study too which remains to be explored. Two times higher incidence of PONV in the N_2_ O group of the present study is not supported by all. A large randomized blind study did not find any significant association between the use of N_2_ O and PONV.[5]

This interesting study has a few limitations. The sample size is small and there is no statistically significant difference between groups when patients with severe distention are compared. Objective measurement of the bowel diameter would have been better to estimate bowel distention. At the same time study cohort was hand-assisted laparoscopic donors which may not be applicable for pure laparoscopic surgery. This study does generate considerable interest and larger randomized controlled trials with more objective parameters may be conducted to further clarify the issues.
